# Symbolic Encoding Methods with Entropy-Based Applications to Financial Time Series Analyses

**DOI:** 10.3390/e25071009

**Published:** 2023-06-30

**Authors:** Joanna Olbryś, Natalia Komar

**Affiliations:** Faculty of Computer Science, Bialystok University of Technology, Wiejska 45a, 15-351 Białystok, Poland

**Keywords:** symbolic time series analysis (STSA), symbol–sequence histogram, modified Shannon entropy, stock market, informational efficiency, extreme event

## Abstract

Symbolic encoding of information is the foundation of Shannon’s mathematical theory of communication. The concept of the informational efficiency of capital markets is closely related to the issue of information processing by equity market participants. Therefore, the aim of this comprehensive research is to examine and compare a battery of methods based on symbolic coding with thresholds and the modified Shannon entropy in the context of stock market efficiency. As these methods are especially useful in assessing the market efficiency in terms of sequential regularity in financial time series during extreme events, two turbulent periods are analyzed: (1) the COVID-19 pandemic outbreak and (2) the period of war in Ukraine. Selected European equity markets are investigated. The findings of empirical experiments document that the encoding method with two 5% and 95% quantile thresholds seems to be the most effective and precise procedure in recognizing the dynamic patterns in time series of stock market indices. Moreover, the Shannon entropy results obtained with the use of this symbolic encoding method are homogenous for all investigated markets and unambiguously confirm that the market informational efficiency measured by the entropy of index returns decreases during extreme event periods. Therefore, we can recommend the use of this STSA method for financial time series analyses.

## 1. Introduction

The use of proper symbolic encoding of information is a foundation of the mathematical theory of communication proposed by Shannon in 1948 [1]. Daw et al. [2] indicated that data analysis tools referred to as *symbolization* or *symbolic time series analysis (STSA)* involve the transformation of raw time series data into a series of discretized symbols that are processed to extract information about the generating process. Among others, Buhlmann [3] and Schittenkopf et al. [4] emphasized that a discretization strategy transforming real data time series into symbolic streams is an effective complexity reduction tool. However, there is no general rule for locating an optimal partition for actual data [5], and therefore many symbolic encoding procedures have been proposed in the literature and the results are not homogenous [2,3,6,7,8,9,10,11,12,13].

The well-known concept of informational efficiency of capital markets, which is strictly connected with the Efficient Market Hypothesis (EMH) [14], is closely related to the issue of information processing and depends on the representation of information by equity market participants. Specifically, the EMH defines an efficient market as one in which new information is quickly and correctly reflected in current security prices [15]. The traditional taxonomy of information sets distinguishes between three forms of informational efficiency: (1) weak-form efficiency, (2) semi-strong-form efficiency, and (3) strong-form efficiency. Weak-form efficiency means that the information set includes only the history of prices or returns. Semi-strong-form efficiency denotes that the information set includes all publicly available information. The final one, strong-form efficiency, means that the information set includes all information known to any market participant (see, e.g., [16]). Gulko [17] emphasized that the idea of market efficiency is linked to the concept of entropy. The author proposed the so-called entropic market hypothesis, which states that the entropy maximization may be a basic property of efficient pricing.

In their seminal paper, Shannon [1] documented that information content could be measured by entropy. Generally speaking, communication systems can be roughly classified into three main categories: discrete, continuous, and mixed. A typical case of a discrete system of communication is telegraphy, since both the message and the signal are a sequence of discrete symbols. In such a meaning, each discrete time series constitutes a discrete source of information that can be encoded with the use of symbols [1]. The Shannon entropy definition is grounded on symbolic representation of the information with the respective estimated probabilities. According to the literature, the modified Shannon entropy approach based on symbolic encoding with thresholds is especially useful in assessing market (in)efficiency in terms of sequential regularity in financial time series during extreme event periods [3].

Within turbulent periods, regularity in financial time series increases in terms of the existence of patterns in stock and index returns. For instance, Risso [10] documented that market trends (both up and down) that are common during extreme event periods usually reduce the entropy of daily financial time series due to more frequent repeated patterns. Specifically, stock market crashes create declining trends in financial time series, which reduce the entropy but increase time series regularity. In general, the chances for price prediction rise within a crisis and economic downturns. To sum up, various methods based on entropy enable investors to evaluate the aforementioned problems (see, e.g., [18] and the references therein).

The main research question of this study can be formulated as follows: which STSA method is the most effective one in entropy-based applications in financial markets, especially in the context of informational market efficiency? Therefore, the aim of this comprehensive research is to evaluate several methods based on the modified Shannon entropy and a symbolic representation of discrete time series in financial market analyses. In order to answer the research question, two turbulent periods are analyzed: (1) the COVID-19 pandemic outbreak and (2) the period of war in Ukraine. Fifteen selected European equity markets are investigated. As the analyzed sample periods are not long, the methods that allow for assessing market (in)efficiency within long-time periods (such as the Hurst exponent [19]) are not appropriate in this case.

The added value of this study is derived from the novel methodological and empirical findings that have not been presented in the literature thus far. The contribution is twofold. First of all, to the authors’ knowledge, this is the first comprehensive piece of research that examines and compares a battery of symbolic encoding methods with thresholds in empirical analyses concerning the informational efficiency of financial markets. Moreover, after symbolization, the dynamic structure in real data is recognized by symbol sequences and symbol sequence histograms of relative frequencies, which provide a convenient way to observe possible patterns in time series. The findings document that the encoding method with two 5% and 95% quantile thresholds seems to be the most effective and precise procedure in assessing dynamic patterns in time series of stock market indices. Therefore, we can recommend the use of this STSA method for financial time series analyses.

Second, the research hypothesis that the market informational efficiency measured by the modified Shannon entropy of daily index returns decreases during extreme event periods is assessed. To examine this hypothesis, changes in entropy values for the pre-turbulent and turbulent periods are estimated. The comparative results are homogenous for both pairs of the pre-event and event sub-periods and they confirm that there is no reason to reject the research hypothesis. The results support the evidence that stock market efficiency measured by entropy decreases during extreme events as the sequential regularity in time series increases in such cases. This conclusion is important for academics and practitioners and it is consistent with the existing literature which documents that turbulent periods are usually found to reduce the entropy of financial markets (e.g., [18,20,21,22,23]).

The rest of this study is organized as follows. Section 2 presents a brief literature review. Section 3 describes the methodological background concerning symbolic encoding methods as well as the modified Shannon entropy approach based on symbolic representations of time series. Section 4 contains real data descriptions. Section 5 presents the experimental studies and compares empirical results on the European stock markets. The last section summarizes and discusses the main findings and indicates some further research directions. The paper is supplemented with three appendixes.

## 2. Brief Literature Review

The literature contains several studies that utilize entropy-based procedures with symbolic encoding in various applications in financial market analyses. However, the number of these studies is rather limited. For instance, Brida and Punzo [5] constructed an artificial economy of the Italian macro-regions and focused on the STSA approach and the modified Shannon entropy to analyse the six-regime dynamics. Risso [10] investigated the daily informational efficiency of five stock markets by using the local entropy and a symbolic time series analysis with one threshold. Mensi et al. [24] evaluated the time-varying degree of the weak-form efficiency of the crude oil market using the modified Shannon entropy and the STSA approach. Sensoy et al. [12] assessed the strength and direction of information flow between exchange rates and stock prices in emerging markets by the effective transfer entropy and symbolic encoding method with two thresholds. Risso [11] applied a symbolic time series analysis with one threshold and the Shannon entropy in order to measure and rank the informational efficiency of twenty developed and emerging stock markets within the world. Mensi et al. [25] examined two worldwide crude oil benchmarks and used the Shannon entropy based on the STSA procedure to rank the market-level efficiency. Oh et al. [26] explored the degree of uncertainty in the return time series of several market indices based on the Shannon entropy, which incorporated the contribution of possible patterns. Ahn et al. [6] used the Shannon entropy based on symbolic encoding with one threshold to analyse the effect of stock market uncertainty on economic fundamentals in China. Shternshis et al. [13] proposed a computational methodology to estimate the Shannon entropy of high-frequency data to study the informational efficiency of Exchange Traded Funds (ETF). The authors considered symbolic encoding methods with one and two thresholds. Olbrys and Majewska [9] applied two different STSA procedures with one threshold and the Shannon entropy to rank European stock markets’ informational (in)efficiency during the COVID-19 pandemic. In a recent paper, Brouty and Garcin [27] determined the amount of information contained in the time series of price returns by using the Shannon entropy applied to symbolic representations of time series.

Another strand of the literature explores the topic of stock market efficiency during turbulent periods with the use of various entropy-based methods. For instance, Wang and Wang [22] documented that the informational efficiency of the S&P 500 index substantially decreased during the COVID-19 extreme event. Ozkan [21] investigated six developed equity markets during the COVID-19 pandemic outbreak and obtained that all markets deviate from market efficiency within this extreme event period. Ortiz-Cruz et al. [20] indicated (based on the multi-scale Approximate Entropy procedure) that returns from crude oil markets were less uncertain during economic downturns. Olbrys and Majewska [18] utilized a different approach, i.e., the Sample Entropy algorithm, to estimate the sequential regularity and entropy of the daily time series of 36 stock market indices within two extreme event periods: (1) the Global Financial Crisis in 2007–2009 and (2) the COVID-19 pandemic outbreak in 2020–2021. In general, the empirical results support the hypothesis that the regularity in financial time series usually increases, while the entropy and informational efficiency of stock markets usually decrease during various turbulence periods due to the existence of patterns in returns. In this context, Billio et al. [28] proposed an entropy-based early warning indicator for systematic risk and documented the forecasting ability of entropy measures in predicting banking crises.

## 3. Methodological Background

This section presents the theoretical background concerning the modified Shannon entropy approach based on symbolic encoding with thresholds. As mentioned in the Introduction, this method is especially useful in assessing the regularity of discrete financial time series during extreme event periods [3].

In this research, the time series of stock market indices are investigated. The returns of indices are calculated as daily logarithmic rates of return:

(1) $$r_{t}=lnP_{t}-lnP_{t-1},$$

where $P_{t}$ is the closing value of the particular market index on day *t*.

### 3.1. Symbolic Encoding with One Threshold

Symbolic time series analyses (STSA) are utilized in many applications. The main idea is that the values of given discrete time series data are transformed into a finite set of symbols, thus obtaining a finite string. This operation is just a translation into a finite precision language [5]. Schittenkopf et al. [4] showed that the discretization of financial time series can effectively filter the data and reduce the noise. Ahn et al. [6] pointed out that an STSA allows to capture time-varying patterns in stock returns by transforming the real data into a limited number of symbols which reflect the dynamic rise–fall pattern of several consecutive returns. Risso [29] reviewed various applications of the STSA methods in social sciences. Letellier [30] emphasized that symbolic sequence analyses are a useful tool for characterizing any kind of dynamical behaviour with symbols, and a so-called threshold-crossing technique could be used. We adapt Letellier’s definition of a sequence of symbols for returns of stock market indices (Definition 1):

**Definition 1.**  *A sequence $\left\{s_{t}\right\}$ of symbols is defined according to:*

(2) $$s_{t}=\left\{\begin{array}{cc} 0 & if\;r_{t}\leq r_{c}\\ 1 & if\;r_{t}>r_{c} \end{array}\right.$$

*where $r_{c}$ is the critical point (threshold) of return time series $\left\{r_{t}\right\}$.*

In the literature, various threshold values $r_{c}$ are taken into consideration, for instance:

1. Method 1: $r_{c}=0$ [6,9,10,24];
2. Method 2: $r_{c}=mean$ [9,11];
3. Method 3: $r_{c}=median$ [8].

The finite set A={0,1} of possible n=2 symbols is called an alphabet, while each subset of a sequence of symbols is called a word [1,2,5,31]. A sequence of consecutive returns is symbolized as a sequence of 0 s and 1 s [6].

### 3.2. Symbolic Encoding with Two Thresholds

To code the same raw data, one can use different discrete coding alphabets corresponding to different levels of discretization. In the case of two thresholds, the set A={0,1,2} of possible symbols is an alphabet. The alphabet size is equal to n=3. Therefore, a sequence of consecutive returns is symbolized as a sequence of 0 s, 1 s and 2 s. Daw et al. [2] emphasized that the binary symbolization for n=2 is convenient in many cases, but higher values of n>2 correspond to an increasingly refined discrimination of measurement details.

The definition of a sequence of symbols for returns of stock market indices in the case of two thresholds is given as follows (Definition 2):

**Definition 2.**  *A sequence $\left\{s_{t}\right\}$ of symbols is defined according to:*

(3) $$s_{t}=\left\{\begin{array}{cc} 0 & if\;r_{t}\leq \theta_{1}\\ 1 & if\;\theta_{1}<r_{t}\leq \theta_{2}\\ 2 & if\;r_{t}>\theta_{2} \end{array}\right.$$

*where $\theta_{1}$ and $\theta_{2}$ are the thresholds of return time series $\left\{r_{t}\right\}$.*

Based on the literature, various thresholds $\theta_{1}$, $\theta_{2}$ are used in empirical research, for instance:

1. Method 1: $\theta_{1}$ is the 5% sample quantile and $\theta_{2}$ is the 95% sample quantile [12];
2. Method 2: $\theta_{1}$ is the 2.5% sample quantile and $\theta_{2}$ is the 97.5% sample quantile [3];
3. Method 3: $\theta_{1}$ and $\theta_{2}$ are the tertiles [13].

### 3.3. Symbolic Sequence Dynamics and Histograms

According to the symbolic dynamics literature, after symbolization, the next step in identification of temporal patterns in time series is the construction of symbol sequences (words). If each possible sequence is represented in terms of a unique identifier, each result creates a new time series referred to as a code series [2]. The choice of a specific decimal number can be arbitrary [6].

Let n>1 be the number of possible symbols and k≥1 be the length of a code sequence (i.e., a word). Hence, there are $n^{k}$ paths with *k* symbols that occur in the given symbolic data sequence. For instance, in the case of n=2 and k=2, the number of possible patterns is equal to $n^{k}=2^{2}=4$ permutations, and all possible words are {(1,1),(1,0),(0,1),(0,0)}. These words can be represented by natural numbers {1,2,3,4} [5].

The temporal structure of observed data is revealed by the relative frequency of each possible symbol sequence (word). The observed dynamics can be described by a *k*-histogram of relative frequencies. The empirical distribution presented in a symbol–sequence histogram allows for a comparison of coded sequences. Generally speaking, direct visual presentation of the frequencies with symbol–sequence histograms provides a convenient way for observing and determining possible patterns in time series [2,5,26].

### 3.4. The Modified Shannon Entropy Approach Based on the Symbolic Representation of Time Series

As mentioned in the Introduction, entropy is a widely used measure that summarizes the information content of a probability distribution. Specifically, the Shannon information entropy [1] quantifies the expected value of information contained in a discrete distribution. The Shannon entropy of *k*-th order (Definition 3) is an information theoretic measure for symbol–sequence frequencies.

**Definition 3.**  *The Shannon entropy of k-th order, H(k), is defined according to:*

(4) $$H\left(k\right)=-\sum_{i}p_{i}\cdot log_{2}\left(p_{i}\right)$$

*where $p_{i}$ is the probability of finding the i-th sequence of length k.*

The probability $p_{i}$ is approximated by the number of times the *i*-th sequence is found in the original symbolic string divided by the number of all non-zero sequences of length *k* [5]. This means that $p_{i}$ is calculated based on the histogram of symbol sequence frequencies.

For increasingly longer sequences from a finite-length time series, the entropy given by Definition 3 tends to be underestimated [2]. Therefore, following Brida and Punzo [5], we use the definition of the modified Shannon entropy $H_{s}\left(k\right)$ based on the symbolic representation of time series (Definition 4). The $H_{s}\left(k\right)$ is a normalized form of the Shannon entropy H(k) (Definition 3).

**Definition 4.**  *The modified (normalized) Shannon entropy $0\leq H_{s}\left(k\right)\leq 1$ based on symbolic representations of time series is defined according to:*

(5) $$H_{s}\left(k\right)=\frac{-1}{log_{2}N}\cdot \sum_{i}p_{i}\cdot log_{2}\left(p_{i}\right)$$

*where N is the total number of observed sequences of length k with non-zero frequency, i is the index of a sequence and $p_{i}$ is the probability of finding the i-th sequence of length k. It is assumed that $0\cdot log_{2}0=0$.*

## 4. Real Data Description

This section describes the real datasets that have been used in empirical research. In order to ensure the coherence and comparability of empirical findings, both datasets include daily observations of the main stock market indices for the same fifteen European countries that have been chosen in the context of the Russian invasion of Ukraine. These countries are France, the United Kingdom, Germany, Finland, Norway, Turkey and the so-called ‘Bucharest 9’ (NATO Eastern flank states, i.e., Poland, Hungary, Czechia, Romania, Bulgaria, Lithuania, Estonia, Latvia and Slovakia). The choice of the selected countries can be justified as follows: (1) France, the United Kingdom, Germany and Turkey have taken an active part in diplomatic efforts concerning the Russian–Ukrainian conflict, (2) Finland and Norway border Russia and (3) all members of the so-called ‘Bucharest 9’ NATO Eastern flank states were either part of the former Soviet Union (USSR) or members of the defunct Soviet-led Warsaw Pact.

### 4.1. The COVID-19 Pandemic Outbreak

The first four-year sample comprises the two-year pre-COVID-19 pandemic period (from January 2018 to December 2019) and the two-year COVID-19 pandemic period (from January 2020 to December 2021). Since there is no unanimity in determining the COVID-19 pandemic period among researchers, in this study it is assumed that this period comprised two years (2020–2021), since on 30 January 2020, the COVID-19 outbreak was declared as a Public Health Emergency of International Concern by the World Health Organization (WHO), while on 11 March 2020, the WHO officially declared the COVID-19 outbreak to be a global pandemic [32].

Table 1 includes brief information about the analyzed indices in the order of decreasing value of market capitalization (in EUR billion) on 31 December 2020, as well as the summarized statistics for the daily logarithmic rates of return within the entire first sample period and two investigated sub-periods.

### 4.2. The War in Ukraine

The second two-year sample comprises the one year pre-war period in Ukraine (from 24 February 2021 to 23 February 2022) and a one-year war period in Ukraine (from 24 February 2022 to 24 February 2023). Analogous to Table 1, the subsequent Table 2 presents brief information about the analyzed indices in the same order and the summarized statistics for the daily logarithmic rates of return within the entire second sample period and two investigated sub-periods.

## 5. Empirical Experiments

This section presents various empirical experiments concerning symbolic encoding with thresholds and entropy-based comparative analyses in the context of sequential regularity in financial time series. The selected fifteen European stock markets are investigated within the turbulent periods including the COVID-19 pandemic outbreak and the war in Ukraine.

Computations were conducted with a dedicated program. To perform the calculations and generate graphs, the Jupyter Notebook—a web-based interactive computing platform—and the pandas, numpy, math, matplotlib and itertools libraries were used. Jupyter (formerly known as IPython Notebook) allows to create documents that include the code and visualizations, while the libraries enable users to process data and generate graphs efficiently.

The findings of real data experiments document that the modified Shannon entropy based on the encoding method with two 5% and 95% quantile thresholds seems to be the most effective explicit procedure in assessing the dynamic patterns in the time series of stock market indices. This method evaluates extreme returns during turbulent periods much more appropriately than other methods, and the empirical results are especially homogenous for all investigated equity markets within all analyzed sub-periods.

### 5.1. Symbolic Dynamic Patterns in Financial Time Series

As mentioned in Section 3.3, direct identification of symbolic dynamic patterns in time series consists of two steps. The first step encompasses symbolic encoding with one or two thresholds based on Definition 1 or Definition 2, respectively. The next step is the construction of symbol sequences (words). Each possible sequence is represented in terms of a unique identifier (code) given by a natural number. Table 3 reports the number of all possible sequences (words) in the case of both definitions.

**Figure 1:**
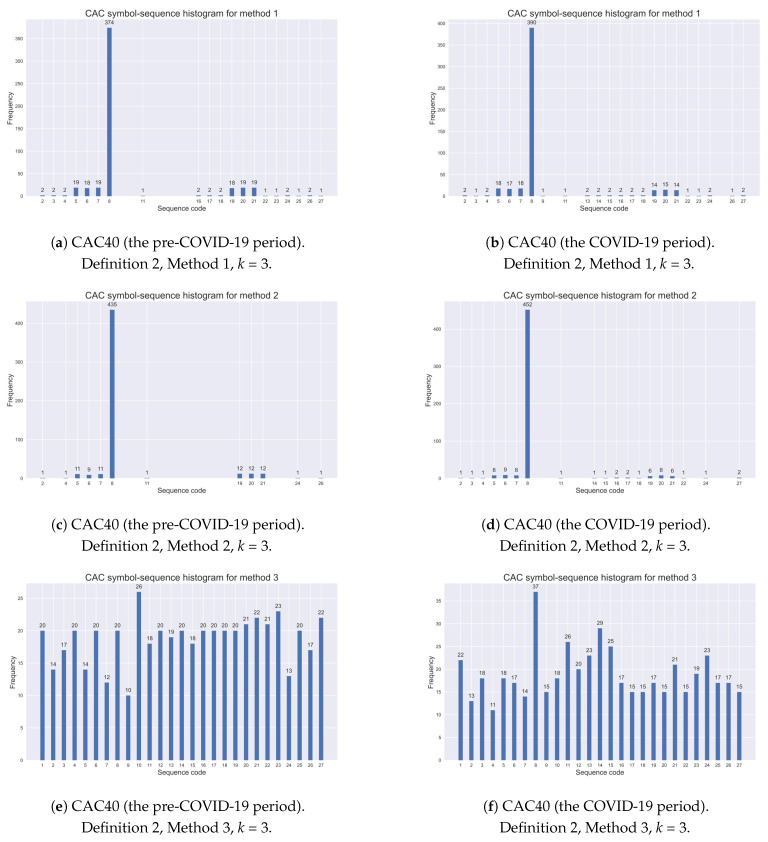
Comparative analysis of symbol–sequence histograms based on Definition 2 and three different STSA methods with two thresholds for the CAC40 (France) index: (**a**) the pre-COVID-19 period, Method 1, (**b**) the COVID-19 period, Method 1, (**c**) the pre-COVID-19 period, Method 2, (**d**) the COVID-19 period, Method 2, (**e**) the pre-COVID-19 period, Method 3, (**f**) the COVID-19 period, Method 3.

**Figure 2:**
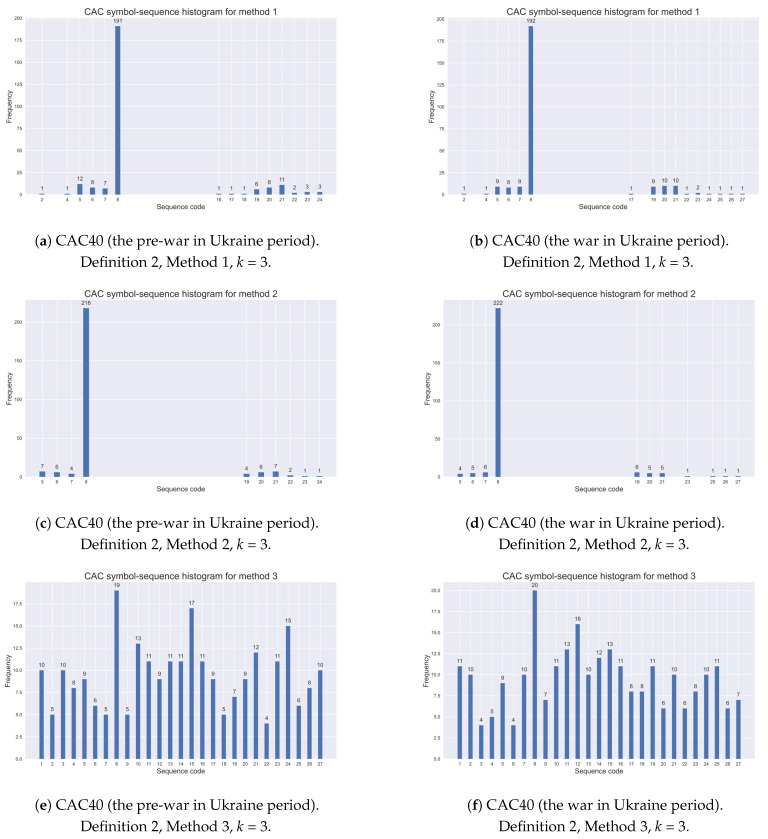
Comparative analysis of symbol–sequence histograms based on Definition 2 and three different STSA methods with two thresholds for the CAC40 (France) index: (**a**) the pre-war in Ukraine period, Method 1, (**b**) the war in Ukraine period, Method 1, (**c**) the pre-war in Ukraine period, Method 2, (**d**) the war in Ukraine period, Method 2, (**e**) the pre-war in Ukraine period, Method 3, (**f**) the war in Ukraine period, Method 3.

Table 4 exemplifies the assigned codes of all possible sequences in the case of the alphabet A={0,1,2} (n=3) and the k=3 length of a code sequence ($n^{k}=3^{3}=27$ natural numbers). Sequence No. 8 (i.e., (1,1,1)) is marked in bold as the one most frequently observed (see Table 5 and Table 6). Moreover, the sequence codes reported in Table 4 are visible in Figure 1 and Figure 2 (Section 5.2) and Figure A2 and Figure A3 (Appendix B).

In this research, three different numbers (k=3,4,5) are utilized as the length of a code sequence. The amount of all calculations is large; thus, only selected results are displayed in this paper. The remaining empirical findings are available upon request.

**Figure A1:**
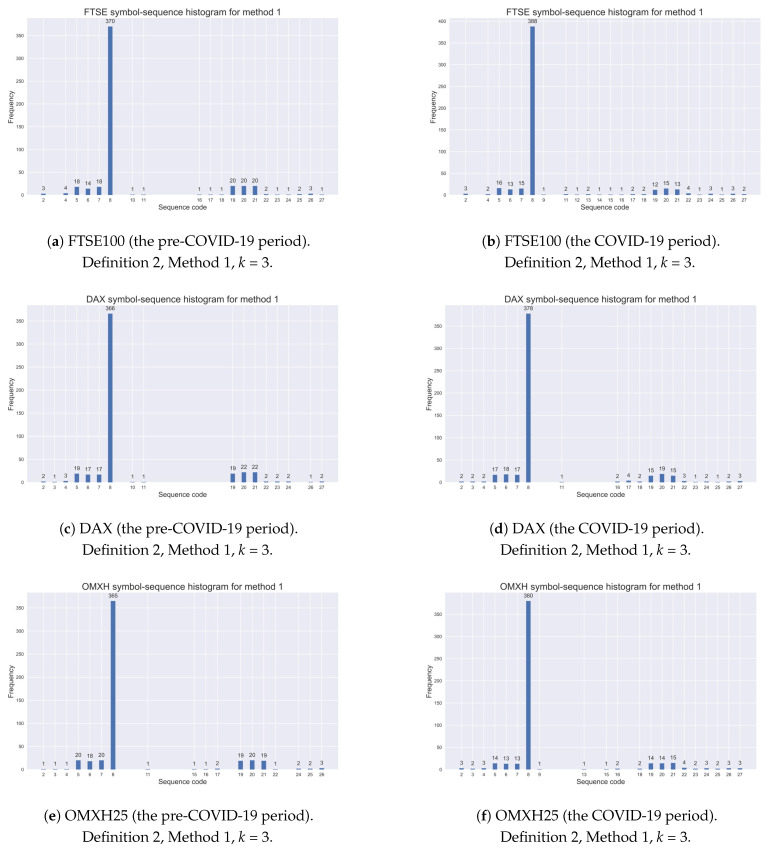
Comparative analysis of symbol–sequence histograms based on Definition 2 and Method 1 with two thresholds (5% and 95%): (**a**) FTSE100 (U.K.)—the pre-COVID-19 period, (**b**) FTSE100 (U.K.)—the COVID-19 period, (**c**) DAX (Germany)—the pre-COVID-19 period, (**d**) DAX (Germany)—the COVID-19 period, (**e**) OMXH25 (Finland)—the pre-COVID-19 period, (**f**) OMXH25 (Finland)—the COVID period.

**Figure A2:**
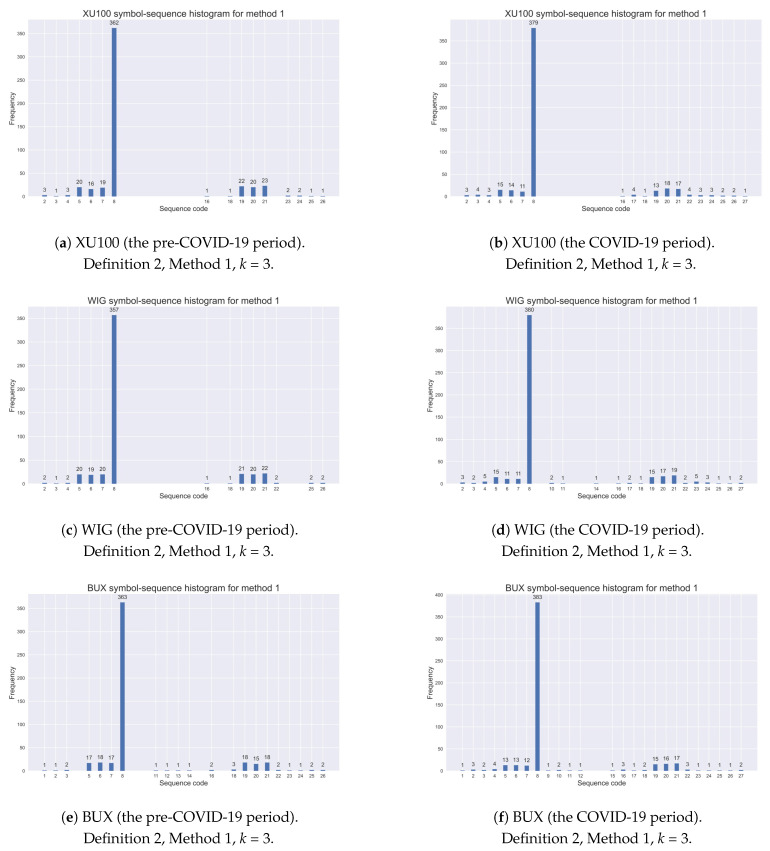
Comparative analysis of symbol–sequence histograms based on Definition 2 and Method 1 with two thresholds (5% and 95%): (**a**) XU100 (Turkey)—the pre-COVID-19 period, (**b**) XU100 (Turkey)—the COVID-19 period, (**c**) WIG (Poland)—the pre-COVID-19 period, (**d**) WIG (Poland)—the COVID-19 period, (**e**) BUX (Hungary)—the pre-COVID-19 period, (**f**) BUX (Hungary)—the COVID period.

**Figure A3:**
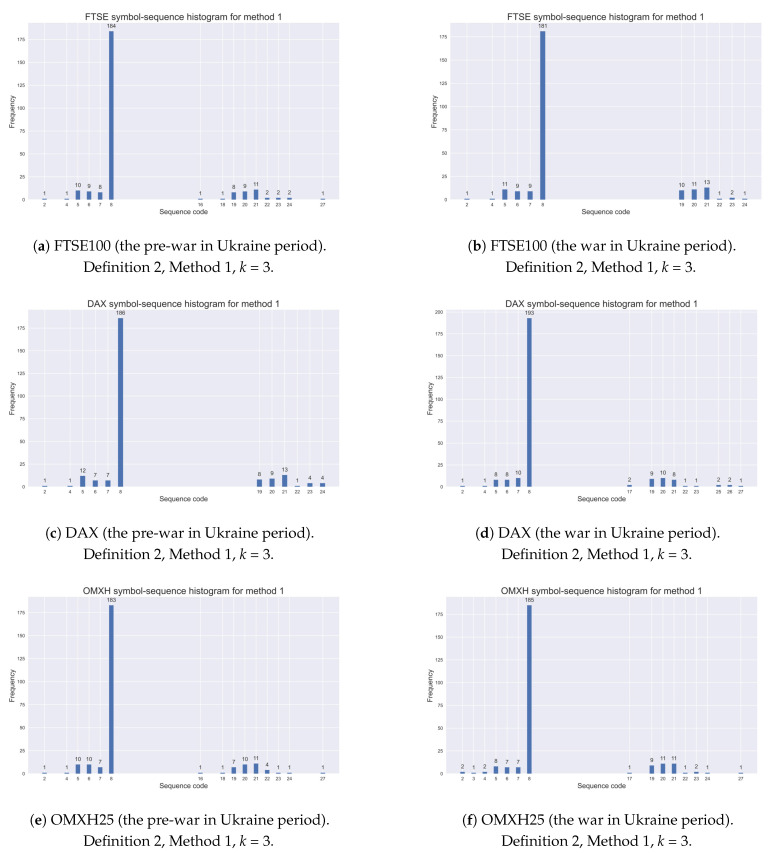
Comparative analysis of symbol–sequence histograms based on Definition 2 and Method 1 with two thresholds (5% and 95%): (**a**) FTSE100 (U.K.)—the pre-war in Ukraine period, (**b**) FTSE100 (U.K.)—the war in Ukraine period, (**c**) DAX (Germany)—the pre-war in Ukraine period, (**d**) DAX (Germany)—the war in Ukraine period, (**e**) OMXH25 (Finland)—the pre-war in Ukraine period, (**f**) OMXH25 (Finland)—the war in Ukraine period.

**Figure A4:**
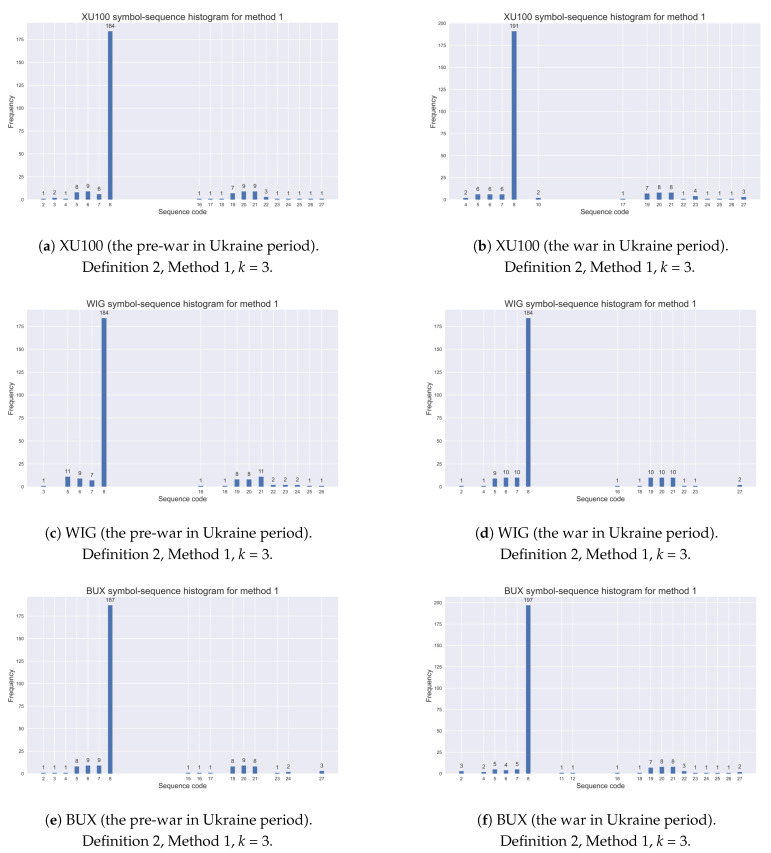
Comparative analysis of symbol–sequence histograms based on Definition 2 and Method 1 with two thresholds (5% and 95%): (**a**) XU100 (Turkey)—the pre-war in Ukraine period, (**b**) XU100 (Turkey)—the war in Ukraine period, (**c**) WIG (Poland)—the pre-war in Ukraine period, (**d**) WIG (Poland)—the war in Ukraine period, (**e**) BUX (Hungary)—the pre-war in Ukraine period, (**f**) BUX (Hungary)—the war in Ukraine period.

Table 5 and Table 6 document that dynamic patterns of symbolic encoding with two thresholds (Definition 2, Method 1: the 5% and 95% sample quantiles) for sequences of length k=3 and k=4 within the pre-event and event periods are homogenous for all fifteen analyzed stock market indices. The results reported in these tables confirm that the sequences (1,1,1) (k=3) and (1,1,1,1) (k=4) are the most frequently observed. These sequences mean that, respectively, three or four successive daily stock index returns are not extremely high or low, but they lie between the sample quantile thresholds, i.e., $\theta_{1}=5\%$ and $\theta_{2}=95\%$ (Definition 2). The percentage numbers of sequences (1,1,1) (k=3) and (1,1,1,1) (k=4) vary between 66.1% and 79.3% (Table 5) and between 62.5% and 79.0% (Table 6). The empirical findings for the (1,1,1) sequence (k=3) are illustrated by the appropriate histograms of relative frequencies for selected indices in Figure A1, Figure A2, Figure A3 and Figure A4 (Appendix B). Moreover, the additional comparative results for k=5 and the sequence (1,1,1,1,1) are reported in Table A1 (Appendix A).

### 5.2. Symbol–Sequence Histograms

As pointed out in Section 3.3, a dynamic structure in real data can be expressed by the relative frequency of each possible symbol sequence. The observed dynamics can be illustrated by a *k*-histogram of relative frequencies. Therefore, in this subsection, selected histograms are presented. For comparison, Figure 1 shows symbol–sequence histograms (k=3) based on Definition 2 and three different STSA methods with two thresholds for the CAC40 (France) index, within the pre-COVID-19 and COVID-19 periods, respectively. Furthermore, Figure 2 exemplifies the appropriate histograms for the same index within the pre-war and war periods in Ukraine.

The evidence shows that Method 1 with two 5% and 95% quantile thresholds specifies extreme returns during turbulent periods more accurately than the other two encoding methods with two thresholds. Method 2 (with two 2.5% and 97.5% quantile thresholds) is too restrictive, while Method 3 (with sample tertiles as the thresholds) collects index returns similarly to the methods with one threshold, given by Definition 1. This observation was commented on in the previous subsection.

The additional Figure A1, Figure A2, Figure A3 and Figure A4 (Appendix B) further express comparative analyses of symbol–sequence histograms based on Definition 2 and Method 1 with two thresholds (5% and 95%) within two pairs of turbulent periods: (1) the pre-COVID-19 and the COVID-19 pandemic periods and (2) the pre-war and war periods in Ukraine.

### 5.3. The Modified Shannon Entropy Comparative Results: The COVID-19 Pandemic Outbreak

In this subsection, the comparative entropy results during the pre-COVID-19 and COVID-19 periods for the fifteen analyzed stock market indices are presented and discussed.

Table 7 includes the findings for the modified Shannon entropy based on three different methods of symbolic encoding with one threshold given by Definition 1 (for sequences of length k=3). The columns entitled ‘Change’ report changes in the Shannon entropy before and during the COVID-19 pandemic period. The down arrows show an entropy decrease, while the up arrows illustrate an entropy increase. As one can observe, the results are rather mixed and heterogenous, and they are not in line with expectations, since the literature documents that the market informational efficiency measured by entropy of index returns usually decreases during extreme event periods [18,20,21,22,23]. Therefore, there is no reason to recommend the use of encoding methods with one threshold for financial time series analyses within extreme event periods.

Table 8 contains the empirical results for the modified Shannon entropy based on three different methods of symbolic encoding with two threshold given by Definition 2 (for sequences of length k=3). It is evident that the modified Shannon entropy values given by Definition 4 depend on the choice of the encoding procedure. This is rather obvious since the lower entropy values determined by Definition 4 are directly connected to a higher level of regularity in time series, expressed by symbol sequences. Conversely, higher entropy values represent a lower level of regularity. Therefore, the entropy values obtained from Method 2 are the lowest, while the results from Method 3 are the highest. These results are associated with the demonstration graphs presented in Figure 1 and Figure 2 (Section 5.2). It is worthwhile noting that the results are homogenous for all investigated stock market indices.

### 5.4. Modified Shannon Entropy Comparative Results: The War in Ukraine

Similarly to the previous subsection, this subsection describes and discusses the modified Shannon entropy comparative results during the pre-war and war periods in Ukraine for the fifteen analyzed stock market indices. The general conclusions are very similar.

Firstly, the results reported in Table 9 (the modified Shannon entropy based on three different methods of symbolic encoding with one threshold given by Definition 1, k=3) are diverse and ambiguous, and they are not in line with expectations. Hence, we cannot advocate the use of STSA methods with one threshold in financial time series analyses within turbulent periods.

Secondly, the findings displayed in Table 10 (the modified Shannon entropy based on three different methods of symbolic encoding with two thresholds given by Definition 2, k=3) are much better compared to those in Table 9, specifically for Method 1 (with two 5% and 95% quantile thresholds). In the case of this method, the pronounced decrease in the modified Shannon entropy for all investigated stock markets is visible in the fourth column (‘Change’) in Table 10. The obtained results are much more homogenous compared to those in Table 9, especially for Method 1 (the fourth column). The up arrows are rare and they are visible only in the case of three markets (i.e., the U.K., Poland and Estonia). Hence, we can recommend the use of Method 1 with two 5% and 95% quantile thresholds in assessing stock market efficiency.

It is important to note that the obtained results decidedly support the research hypothesis. Therefore, we can assert that the recommendation of the use of the STSA method with two 5% and 95% quantile thresholds is well founded.

Furthermore, additional comparative findings of Method 1 (the 5% and 95% sample quantiles) for sequences of length k=4 and k=5 within the pre-event and event periods for the fifteen analyzed stock market indices are reported in Table A4 and Table A5 (Appendix C). The obtained results indicate that the choice of the sequence length *k* is a minor issue as the results for k=4 and k=5 are very similar to those for k=3. However, the visualization of the results by *k*-histograms is much more difficult for k=4 and k=5, as the total number of possible sequences is large (see Table 3).

## 6. Conclusions

The purpose of this empirical study was to investigate and compare various methods based on the symbolic representation of discrete time series and the modified Shannon entropy in assessing stock market informational efficiency in terms of sequential regularity in financial time series. Fifteen European stock markets within two extreme event periods (i.e., the COVID-19 pandemic outbreak and the war in Ukraine) were analyzed. The markets were selected in the context of the Russian invasion of Ukraine. To capture the sequential dynamics in daily time series of equity market indices, changes in the Shannon entropy values before and during the particular extreme event were calculated and compared. The research hypothesis that the stock market efficiency measured by entropy usually decreases during turbulent periods was examined with the use of six different variants of STSA methods.

The research contribution of our paper to the discussion concerning stock market informational efficiency is twofold. Firstly, the most pronounced and consistent empirical results were obtained with the use of the STSA method with two thresholds (the 5% and 95% sample quantiles). This method was the best and most unambiguous in assessing the stock market efficiency measured by the modified Shannon entropy. Moreover, the empirical findings confirmed no reason to reject the proposed research hypothesis, since the entropy of stock market indices visibly decreased during both turbulent periods. This well-justified observation is consistent with the existing literature, and it is the second important contribution of our study.

The obtained comparative findings were especially unambiguous within the pre-COVID-19 and COVID-19 sub-periods. This evidence is rather obvious. It is worth recalling that the European stock markets were affected by the COVID-19 pandemic outbreak at the same time and to a similar extent, as opposed to the influence of the war in Ukraine.

It is worth mentioning that our research relates to the literature concerning the weak form of market informational efficiency, since the used information sets include only the history of index returns. Stock market index returns contain the influence of public information and, during various extreme event periods, all public information is especially important for investors and determines investment decisions. Among others, Lim and Brooks [15] emphasized that the empirical findings of market efficiency are rather heterogenous, as the EMH remains an elusive concept. Generally speaking, the topic is interesting and valid for academics and practitioners, and the recommended STSA method might be used as a useful tool in systems that support investment decisions.

The potential limitations of our research are mainly related to the choice of the investigated European stock markets. However, these limitations are not very significant as this choice is well justified (see Section 4) and the obtained empirical findings are homogenous.

Since the topic of stock market informational efficiency measured by entropy is strictly connected to the problem of market dynamics and volatility, a promising direction for further research could be an extensive assessment of STSA methods that incorporate volatility estimates (e.g., [4]). The motivation for such research can be, for instance, the study conducted by Gradojevic and Caric [33]. The authors emphasize that although volatility and entropy are related measures of market risk and uncertainty, entropy can be more useful in predictive modeling.

## Figures and Tables

**Table 1:** Information about the 15 analyzed stock market indices and the basic statistics for the daily logarithmic rates of return within the entire first sample and two sub-samples.

Country	Index	Market Cap.	The First Sample			Pre-COVID-19			COVID-19		
			N	Mean (in%)	Std. Dev. (in%)	N	Mean (in%)	Std. Dev. (in%)	N	Mean (in%)	Std. Dev. (in%)
France	CAC40	2480.404	1024	0.03	1.27	509	0.02	0.86	515	0.03	1.58
U.K.	FTSE100	2411.490	1011	0.00	1.15	504	0.00	0.77	507	0.00	1.43
Germany	DAX	1870.687	1010	0.02	1.32	501	0.01	0.94	509	0.04	1.61
Finland	OMXH25	289.000	997	0.03	1.15	499	0.01	0.86	498	0.05	1.38
Norway	OSEAX	273.141	1001	0.04	1.18	497	0.03	0.90	504	0.05	1.41
Turkey	XU100	194.491	1000	0.05	1.51	499	−0.01	1.35	501	0.10	1.66
Poland	WIG	145.379	997	0.01	1.24	494	−0.02	0.89	503	0.04	1.51
Hungary	BUX	22.908	992	0.03	1.28	489	0.03	0.98	503	0.02	1.52
Czechia	PX	21.797	999	0.03	0.98	498	0.01	0.62	501	0.05	1.24
Romania	BET	20.895	998	0.05	1.14	497	0.05	1.04	501	0.05	1.23
Bulgaria	SOFIX	14.505	984	−0.01	0.83	491	−0.04	0.58	493	0.02	1.01
Lithuania	OMXV	12.114	994	0.04	0.73	495	0.02	0.57	499	0.06	0.85
Estonia	OMXT	3.014	1001	0.05	0.90	500	0.00	0.45	501	0.09	1.18
Latvia	OMXR	2.971	991	0.02	1.17	494	0.01	1.07	497	0.04	1.26
Slovakia	SAX	2.648	973	0.02	1.01	482	0.02	0.95	491	0.03	1.07

N denotes the number of sample observations.

**Table 2:** Information about the 15 analyzed stock market indices and the basic statistics for the daily logarithmic rates of return within the entire second sample and two sub-samples.

Country	Index	Market Cap.	The Second Sample			Pre-War in Ukraine			War in Ukraine		
			N	Mean (in%)	Std. Dev. (in%)	N	Mean (in%)	Std. Dev. (in%)	N	Mean (in%)	Std. Dev. (in%)
France	CAC40	2480.404	517	0.04	7.09	258	0.06	2.36	259	0.02	4.73
U.K.	FTSE100	2411.490	504	0.03	4.06	253	0.03	1.71	251	0.02	2.50
Germany	DAX	1870.687	514	0.02	7.58	255	0.02	2.41	259	0.02	4.87
Finland	OMXH25	289.000	502	0.005	7.26	251	0.01	2.48	251	−0.003	4.78
Norway	OSEAX	273.141	507	0.05	6.33	252	0.07	2.20	255	0.03	4.13
Turkey	XU100	194.491	499	0.25	21.41	249	0.12	7.37	250	0.37	13.96
Poland	WIG	145.379	504	0.005	9.75	251	0.04	2.67	253	0.02	5.79
Hungary	BUX	22.908	506	0.01	11.83	252	0.04	2.81	254	0.02	7.96
Czechia	PX	21.797	505	0.06	5.27	251	0.12	1.21	254	0.001	3.70
Romania	BET	20.895	503	0.04	4.97	251	0.10	1.55	252	−0.03	3.40
Bulgaria	SOFIX	14.505	493	0.04	3.40	245	0.08	1.32	248	0.01	2.00
Lithuania	OMXV	12.114	500	0.03	2.33	249	0.03	0.89	251	0.06	0.97
Estonia	OMXT	3.014	506	0.05	5.25	252	0.10	3.28	254	0.00	1.93
Latvia	OMXR	2.971	502	0.003	6.34	248	0.03	1.47	254	0.03	4.36
Slovakia	SAX	2.648	471	−0.02	3.00	249	0.04	1.23	222	−0.08	1.87

Notation as in Table 1.

**Table 3:** The number of all possible sequences (words).

A Code Sequence Length	Definition 1, Alphabet A={0,1}	Definition 2, Alphabet A={0,1,2}
k=3	$2^{3}=8$	$3^{3}=27$
k=4	$2^{4}=16$	$3^{4}=81$
k=5	$2^{5}=32$	$3^{5}=243$

**Table 4:** The codes of all possible sequences (words) for the alphabet A={0,1,2} and k=3.

The Codes of Sequences for the Alphabet A={0,1,2} and k=3
$\begin{array}{ccccccccc} 1 & 2 & 3 & 4 & 5 & 6 & 7 & 8 & 9\\ \left(0,0,0\right) & \left(0,0,1\right) & \left(0,1,0\right) & \left(1,0,0\right) & \left(1,1,0\right) & \left(1,0,1\right) & \left(0,1,1\right) & \left(1,1,1\right) & \left(2,2,2\right)\\ 10 & 11 & 12 & 13 & 14 & 15 & 16 & 17 & 18\\ \left(0,0,2\right) & \left(0,2,0\right) & \left(2,0,0\right) & \left(2,2,0\right) & \left(2,0,2\right) & \left(0,2,2\right) & \left(2,2,1\right) & \left(2,1,2\right) & \left(1,2,2\right)\\ 19 & 20 & 21 & 22 & 23 & 24 & 25 & 26 & 27\\ \left(1,1,2\right) & \left(1,2,1\right) & \left(2,1,1\right) & \left(0,1,2\right) & \left(0,2,1\right) & \left(1,0,2\right) & \left(1,2,0\right) & \left(2,0,1\right) & \left(2,1,0\right) \end{array}$

**Table 5:** Dynamic patterns of symbolic encoding with two thresholds (Method 1: the 5% and 95% sample quantiles) for sequences of length k=3 and k=4 within the pre-COVID-19 and COVID-19 periods (for the 15 analyzed stock market indices).

Country	The Pre-COVID-19 Period				The COVID-19 Period			
	All Sequences for *k* = 3	The Sequence (1,1,1)	All Sequences for *k* = 4	The sequence (1,1,1,1)	All Sequences for *k* = 3	The Sequence (1,1,1)	All Sequences for *k* = 4	The Sequence (1,1,1,1)
France	507	374 (73.8%)	506	344 (68.0%)	513	390 (76.0%)	512	365 (71.3%)
U.K.	502	370 (73.7%)	501	335 (66.9%)	505	388 (76.8%)	504	364 (72.2%)
Germany	499	366 (73.3%)	498	333 (66.9%)	507	378 (74.6%)	506	353 (69.8%)
Finland	497	365 (73.4%)	496	330 (66.5%)	496	380 (76.6%)	495	358 (72.3%)
Norway	495	368 (74.3%)	494	338 (68.4%)	502	384 (76.5%)	501	361 (72.1%)
Turkey	497	362 (72.8%)	496	328 (66.1%)	499	379 (76.0%)	498	353 (70.9%)
Poland	492	357 (72.6%)	491	321 (65.4%)	501	380 (75.8%)	500	352 (70.4%)
Hungary	487	363 (74.5%)	486	332 (68.3%)	501	383 (76.4%)	500	360 (72.0%)
Czechia	496	371 (74.8%)	495	339 (68.5%)	499	383 (76.8%)	498	360 (72.3%)
Romania	495	367 (74.1%)	494	334 (67.6%)	499	391 (78.4%)	498	372 (74.7%)
Bulgaria	489	374 (76.5%)	488	345 (70.7%)	491	383 (78.0%)	490	358 (73.1%)
Lithuania	493	381 (77.3%)	492	356 (72.4%)	497	394 (79.3%)	496	376 (75.8%)
Estonia	498	376 (75.5%)	497	344 (69.2%)	499	389 (78.0%)	498	371 (74.5%)
Latvia	492	374 (76.0%)	491	346 (70.5%)	495	377 (76.2%)	494	349 (70.6%)
Slovakia	480	350 (72.9%)	479	319 (66.6%)	489	374 (76.5%)	488	351 (71.9%)

Notation as in Table 1.

**Table 6:** Dynamic patterns of symbolic encoding with two thresholds (Method 1: the 5% and 95% sample quantiles) for sequences of length k=3 and k=4 within the pre-war in Ukraine and the war periods (for the 15 analyzed stock market indices).

Country	The Pre-War in Ukraine Period				The War in Ukraine Period			
	All Sequences for *k* = 3	The Sequence (1,1,1)	All Sequences for *k* = 4	The Sequence (1,1,1,1)	All Sequences for *k* = 3	The Sequence (1,1,1)	All Sequences for *k* = 4	The Sequence (1,1,1,1)
France	256	191 (74.6%)	255	177 (69.4%)	257	192 (74.7%)	256	176 (68.8%)
U.K.	251	184 (73.3%)	250	167 (66.8%)	249	181 (72.7%)	248	162 (65.3%)
Germany	253	186 (73.5%)	252	171 (67.9%)	257	193 (75.1%)	256	178 (69.5%)
Finland	249	183 (73.5%)	248	169 (68.1%)	249	185 (74.3%)	248	170 (68.5%)
Norway	250	183 (73.2%)	249	166 (66.7%)	253	186 (73.5%)	252	171 (67.9%)
Turkey	247	184 (74.5%)	246	171 (69.5%)	248	191 (77.0%)	247	178 (72.1%)
Poland	249	184 (73.9%)	248	166 (66.9%)	251	184 (73.3%)	250	169 (67.6%)
Hungary	250	187 (74.8%)	249	172 (69.1%)	252	197 (78.2%)	251	186 (74.1%)
Czechia	249	175 (70.3%)	248	155 (62.5%)	252	198 (78.6%)	251	186 (74.1%)
Romania	249	186 (74.7%)	248	170 (68.5%)	250	189 (75.6%)	249	175 (70.3%)
Bulgaria	243	175 (72.0%)	242	158 (65.3%)	246	191 (77.6%)	245	178 (72.7%)
Lithuania	247	181 (73.3%)	246	166 (67.5%)	249	194 (77.9%)	248	183 (73.8%)
Estonia	250	194 (77.6%)	249	181 (72.7%)	252	191 (75.8%)	251	176 (70.1%)
Latvia	246	178 (72.4%)	245	161 (65.7%)	252	199 (79.0%)	251	190 (75.7%)
Slovakia	247	183 (74.1%)	246	166 (67.5%)	220	165 (75.0%)	219	150 (68.5%)

Notation as in Table 1.

**Table 7:** The modified Shannon entropy based on symbolic encoding with one threshold given by Definition 1. The comparative empirical findings of three methods for sequences of length *k* = 3 within the pre-COVID-19 and COVID-19 periods for the 15 analyzed stock market indices.

Country	The Modified Shannon Entropy Based on Symbolic Encoding with One Threshold								
	Definition 1, Method 1, *k* = 3			Definition 1, Method 2, *k* = 3			Definition 1, Method 3, *k* = 3		
	Pre-COVID-19	COVID-19	Change	Pre-COVID-19	COVID-19	Change	Pre-COVID-19	COVID-19	Change
France	0.994	0.988	−0.006 ↓	0.996	0.992	−0.004 ↓	0.999	0.998	−0.001 ↓
U.K.	0.998	0.993	−0.005 ↓	0.998	0.992	−0.006 ↓	0.998	0.994	−0.004 ↓
Germany	0.996	0.994	−0.002 ↓	0.997	0.997	0	0.999	0.996	−0.003 ↓
Finland	0.999	0.992	−0.007 ↓	0.998	0.997	−0.001 ↓	0.999	0.999	0
Norway	0.993	0.996	0.006 ↑	0.998	0.999	0.001 ↑	0.998	0.999	0.001 ↑
Turkey	0.998	0.970	−0.028 ↓	0.998	0.992	−0.006 ↓	0.999	0.999	0
Poland	0.998	0.999	0.001 ↑	0.998	0.999	0.001 ↑	0.998	0.999	0.001 ↑
Hungary	0.997	0.997	0	0.996	0.998	0.002 ↑	0.997	0.998	0.001 ↑
Czechia	0.992	0.989	−0.003 ↓	0.993	0.997	0.004 ↑	0.998	0.997	−0.001 ↓
Romania	0.989	0.979	−0.010 ↓	0.999	0.991	−0.008 ↓	0.999	0.996	−0.003 ↓
Bulgaria	0.991	0.997	0.006 ↑	0.996	0.999	0.003 ↑	0.997	0.999	0.002 ↑
Lithuania	0.994	0.988	−0.006 ↓	0.994	0.997	0.003 ↑	0.995	0.999	0.004 ↑
Estonia	0.999	0.962	−0.037 ↓	0.999	0.979	−0.020 ↓	0.999	0.979	−0.020 ↓
Latvia	0.984	0.983	−0.001 ↓	0.986	0.984	−0.002 ↓	0.987	0.986	−0.001 ↓
Slovakia	0.969	0.908	−0.061 ↓	0.966	0.902	−0.064 ↓	0.969	0.908	−0.061 ↓

Notation as in Table 1.

**Table 8:** The modified Shannon entropy based on symbolic encoding with two thresholds given by Definition 2. The comparative empirical findings of three methods for sequences of length k=3 within the pre-COVID-19 and COVID-19 periods for the 15 analyzed stock market indices.

Country	The Modified Shannon Entropy Based on Symbolic Encoding with Two Thresholds								
	Definition 2, Method 1, *k* = 3			Definition 2, Method 2, *k* = 3			Definition 2, Method 3, *k* = 3		
	Pre-COVID-19	COVID-19	Change	Pre-COVID-19	COVID-19	Change	Pre-COVID-19	COVID-19	Change
France	0.397	0.365	−0.032 ↓	0.280	0.222	−0.058 ↓	0.994	0.989	−0.005 ↓
U.K.	0.398	0.361	−0.037 ↓	0.273	0.240	−0.033 ↓	0.994	0.992	−0.002 ↓
Germany	0.414	0.396	−0.018 ↓	0.297	0.270	−0.027 ↓	0.990	0.984	−0.006 ↓
Finland	0.405	0.376	−0.029 ↓	0.308	0.243	−0.065 ↓	0.992	0.992	0
Norway	0.415	0.376	−0.039 ↓	0.281	0.226	−0.055 ↓	0.989	0.994	0.005 ↑
Turkey	0.423	0.390	−0.033 ↓	0.295	0.272	−0.023 ↓	0.989	0.997	0.008 ↑
Poland	0.433	0.377	−0.056 ↓	0.306	0.240	−0.066 ↓	0.987	0.995	0.008 ↑
Hungary	0.385	0.359	−0.026 ↓	0.266	0.219	−0.047 ↓	0.991	0.995	0.004 ↑
Czechia	0.400	0.368	−0.032 ↓	0.265	0.233	−0.032 ↓	0.994	0.988	−0.006 ↓
Romania	0.407	0.359	−0.048 ↓	0.244	0.216	−0.028 ↓	0.992	0.989	−0.003 ↓
Bulgaria	0.374	0.347	−0.027 ↓	0.248	0.234	−0.014 ↓	0.994	0.994	0
Lithuania	0.358	0.345	−0.013 ↓	0.220	0.232	0.012 ↑	0.993	0.992	−0.001 ↓
Estonia	0.382	0.356	−0.026 ↓	0.257	0.232	−0.025 ↓	0.991	0.978	−0.013 ↓
Latvia	0.416	0.366	−0.050 ↓	0.248	0.227	−0.021 ↓	0.981	0.979	−0.002 ↓
Slovakia	0.431	0.380	−0.051 ↓	0.319	0.261	−0.058 ↓	0.989	0.908	−0.081 ↓

Notation as in Table 1.

**Table 9:** The modified Shannon entropy based on symbolic encoding with one threshold given by Definition 1. The comparative empirical findings of three methods for sequences of length k=3 within the pre-war in Ukraine and war periods for the 15 analyzed stock market indices.

Country	The Modified Shannon Entropy Based on Symbolic Encoding with One Threshold								
	Definition 1, Method 1, *k* = 3			Definition 1, Method 2, *k* = 3			Definition 1, Method 3, *k* = 3		
	Pre-War	War	Change	Pre-War	War	Change	Pre-War	War	Change
France	0.976	0.997	0.021 ↑	0.990	0.997	0.007 ↑	0.999	0.998	−0.001 ↓
U.K.	0.981	0.985	0.004 ↑	0.984	0.987	0.003 ↑	0.988	0.998	0.010 ↑
Germany	0.988	0.996	0.008 ↑	0.989	0.997	0.008 ↑	0.995	0.997	0.002 ↑
Finland	0.991	0.983	−0.008 ↓	0.991	0.983	−0.008 ↓	0.999	0.997	−0.002 ↓
Norway	0.996	0.993	−0.003 ↓	0.999	0.993	−0.006 ↓	0.999	0.993	−0.006 ↓
Turkey	0.954	0.929	−0.025 ↓	0.993	0.997	0.004 ↑	0.998	0.997	−0.001 ↓
Poland	0.992	0.994	0.002 ↑	0.993	0.994	0.001 ↑	0.992	0.996	0.004 ↑
Hungary	0.998	0.988	−0.010 ↓	0.999	0.985	−0.014 ↓	0.999	0.996	−0.003 ↓
Czechia	0.967	0.990	0.023 ↑	0.994	0.988	−0.006 ↓	0.994	0.994	0
Romania	0.970	0.995	0.025 ↑	0.998	0.995	−0.003 ↓	0.998	0.999	0.001 ↑
Bulgaria	0.988	0.998	0.010 ↑	0.996	0.998	0.002 ↑	0.997	0.998	0.001 ↑
Lithuania	0.995	0.977	−0.018 ↓	0.998	0.993	−0.005 ↓	0.999	0.995	−0.003 ↓
Estonia	0.959	0.989	0.030 ↑	0.979	0.991	0.012 ↑	0.980	0.987	0.007 ↑
Latvia	0.975	0.995	0.020 ↑	0.977	0.996	0.019 ↑	0.978	0.996	0.018 ↑
Slovakia	0.825	0.857	0.032 ↑	0.860	0.859	−0.001 ↓	0.825	0.857	0.032 ↑

Notation as in Table 1.

**Table 10:** The modified Shannon entropy based on symbolic encoding with two thresholds given by Definition 2. The comparative empirical findings of three methods for sequences of length k=3 within the pre-war in Ukraine and war periods for the 15 analyzed stock market indices.

Country	The Modified Shannon Entropy Based on Symbolic Encoding with Two Thresholds								
	Definition 2, Method 1, *k* = 3			Definition 2, Method 2, *k* = 3			Definition 2, Method 3, *k* = 3		
	Pre-War	War	Change	Pre-War	War	Change	Pre-War	War	Change
France	0.425	0.412	−0.013 ↓	0.313	0.284	−0.029 ↓	0.978	0.980	0.002 ↑
U.K.	0.435	0.466	0.031 ↑	0.291	0.304	0.013 ↑	0.981	0.987	0.006 ↑
Germany	0.461	0.417	−0.044 ↓	0.321	0.285	−0.036 ↓	0.989	0.988	−0.001 ↓
Finland	0.433	0.426	−0.007 ↓	0.325	0.254	−0.071 ↓	0.988	0.982	−0.006 ↓
Norway	0.451	0.434	−0.017 ↓	0.286	0.358	0.072 ↑	0.981	0.980	−0.001 ↓
Turkey	0.407	0.398	−0.009 ↓	0.269	0.269	0	0.989	0.989	0
Poland	0.431	0.439	0.008 ↑	0.297	0.340	0.043 ↑	0.989	0.988	−0.001 ↓
Hungary	0.416	0.370	−0.046 ↓	0.309	0.237	−0.072 ↓	0.994	0.986	−0.008 ↓
Czechia	0.532	0.361	−0.171 ↓	0.362	0.248	−0.114 ↓	0.984	0.989	0.005 ↑
Romania	0.417	0.405	−0.012 ↓	0.325	0.256	−0.069 ↓	0.983	0.978	−0.005 ↓
Bulgaria	0.464	0.376	−0.088 ↓	0.356	0.230	−0.126 ↓	0.984	0.986	0.002 ↑
Lithuania	0.431	0.369	−0.062 ↓	0.299	0.232	−0.067 ↓	0.986	0.988	0.002 ↑
Estonia	0.383	0.406	0.023 ↑	0.257	0.250	−0.007 ↓	0.975	0.974	−0.001 ↓
Latvia	0.456	0.361	−0.095 ↓	0.328	0.249	−0.079 ↓	0.979	0.979	0
Slovakia	0.438	0.410	−0.028 ↓	0.306	0.300	−0.006 ↓	0.825	0.857	0.032 ↑

Notation as in Table 1.

## Data Availability

The data come from the following web pages: Stooq (https://stooq.pl, 28 February 2023); Nasdaq (http://www.nasdaqomxnordic.com, 28 February 2023).

## Abbreviations

The following abbreviations are used in this manuscript:

STSA	Symbolic Time Series Analysis
EMH	Efficient Market Hypothesis
COVID-19	COVID-19 pandemic

## Appendix A. Symbolic Dynamic Patterns in Financial Time Series: Additional Comparative Results

Table A1 reports the comparative results for k=5 and the sequence (1,1,1,1,1) (Definition 2, Method 1). Analogously to the cases for k=3 and k=4 documented in Table 5 and Table 6, the empirical findings are also homogenous for all investigated stock markets and all sub-periods. The evidence shows that the sequence (1,1,1,1,1) is the most frequent path. The percentage number of this sequence is high and it varies between 55.9% and 72.4%. The sequence (1,1,1,1,1) means that five successive daily stock index returns are not extreme as they lie between the sample thresholds of $\theta_{1}=5\%$ and $\theta_{2}=95\%$.

**Table A1:** Dynamic patterns of symbolic encoding with two thresholds (Method 1: the 5% and 95% sample quantiles) for k=5 and the sequence (1,1,1,1,1) within the pre-event and event periods (for the 15 analyzed stock market indices).

Country	Pre-War in Ukraine		War in Ukraine		Pre-COVID-19		COVID-19	
	All Sequences for *k* = 5	The Sequence (1,1,1,1,1)	All Sequences for *k* = 5	The Sequence (1,1,1,1,1)	All Sequences for *k* = 5	The Sequence (1,1,1,1,1)	All Sequences for *k* = 5	The Sequence (1,1,1,1,1)
France	254	164 (64.6%)	255	161 (63.1%)	505	319 (63.2%)	511	345 (67.5%)
U.K.	249	151 (60.6%)	247	149 (60.3%)	500	305 (61.0%)	503	344 (68.4%)
Germany	251	159 (63.3%)	255	165 (64.7%)	497	301 (60.6%)	505	333 (65.0%)
Finland	247	158 (64.0%)	247	156 (63.2%)	495	302 (61.0%)	494	340 (68.8%)
Norway	248	150 (60.5%)	251	159 (63.3%)	493	311 (63.1%)	500	339 (67.8%)
Turkey	245	161 (65.7%)	246	166 (67.5%)	495	301 (60.8%)	497	333 (67.0%)
Poland	247	151 (61.1%)	249	155 (62.2%)	490	289 (59.0%)	499	329 (65.9%)
Hungary	248	159 (64.1%)	250	177 (70.8%)	485	305 (62.9%)	499	339 (67.9%)
Czechia	247	138 (55.9%)	250	175 (70.0%)	494	308 (62.3%)	497	340 (68.8%)
Romania	247	157 (63.6%)	248	164 (66.1%)	493	304 (61.7%)	497	355 (71.4%)
Bulgaria	241	143 (59.3%)	244	165 (67.6%)	487	316 (64.9%)	489	336 (68.7%)
Lithuania	245	154 (62.9%)	247	172 (69.6%)	491	333 (67.8%)	495	358 (72.3%)
Estonia	248	170 (68.5%)	250	162 (64.8%)	496	313 (63.1%)	497	353 (71.0%)
Latvia	244	145 (59.4%)	250	181 (72.4%)	490	323 (65.9%)	493	321 (65.1%)
Slovakia	245	151 (61.6%)	218	135 (61.9%)	478	291 (60.9%)	487	331 (68.0%)

Notation as in Table 1.

The following Table A2 and Table A3 demonstrate the dynamic patterns of symbolic encoding with two thresholds in the case of Definition 2 and Method 2 (the 2.5% and 97.5% sample quantiles) for sequences of length k=3 and k=4 within the pre-event and event periods. Although the results are unambiguous for all fifteen analyzed stock market indices, they reveal that this STSA method is too restrictive, as the percentage numbers of sequences (1,1,1) (k=3) and (1,1,1,1) (k=4) are very high. These numbers vary between 79.7% and 89.0% (Table A2) and between 76.4% and 90.6% (Table A3). This means that Method 2 with $\theta_{1}=2.5\%$ and $\theta_{2}=97.5\%$ as the thresholds is not very useful, since it favours extremely high or low daily returns and such returns are relatively rare.

**Table A2:** Dynamic patterns of symbolic encoding with two thresholds (Method 2: the 2.5% and 97.5% sample quantiles) for sequences of length k=3 and k=4 within the pre-COVID-19 and COVID-19 periods (for the 15 analyzed stock market indices).

Country	The Pre-COVID-19 Period				The COVID-19 Period			
	All Sequences for *k* = 3	The Sequence (1,1,1)	All Sequences for *k* = 4	The sequence (1,1,1,1)	All Sequences for *k* = 3	The Sequence (1,1,1)	All Sequences for *k* = 4	The Sequence (1,1,1,1)
France	507	435 (85.8%)	506	412 (81.4%)	513	452 (88.1%)	512	441 (86.1%)
U.K.	502	432 (86.1%)	501	409 (81.6%)	505	440 (87.1%)	504	427 (84.7%)
Germany	499	425 (85.1%)	498	404 (81.1%)	507	437 (86.2%)	506	421 (83.2%)
Finland	497	423 (85.1%)	496	401 (80.8%)	496	432 (87.1%)	495	418 (84.4%)
Norway	495	426 (86.1%)	494	407 (82.4%)	502	441 (87.8%)	501	430 (85.8%)
Turkey	497	425 (85.5%)	496	404 (81.5%)	499	430 (86.2%)	498	414 (83.1%)
Poland	492	420 (85.4%)	491	397 (80.9%)	501	437 (87.2%)	500	422 (84.4%)
Hungary	487	420 (86.2%)	486	403 (82.9%)	501	443 (88.4%)	500	432 (86.4%)
Czechia	496	428 (86.3%)	495	409 (82.6%)	499	438 (87.8%)	498	425 (85.3%)
Romania	495	434 (87.7%)	494	418 (84.6%)	499	444 (89.0%)	498	435 (87.3%)
Bulgaria	489	425 (86.9%)	488	407 (83.4%)	491	430 (87.6%)	490	417 (85.1%)
Lithuania	493	438 (88.8%)	492	426 (86.6%)	497	436 (87.7%)	496	423 (85.3%)
Estonia	498	434 (87.1%)	497	415 (83.5%)	499	439 (88.0%)	498	426 (85.5%)
Latvia	492	433 (88.0%)	491	418 (85.1%)	495	435 (87.9%)	494	420 (85.0%)
Slovakia	480	405 (84.4%)	479	382 (79.7%)	489	422 (86.3%)	488	406 (83.2%)

Notation as in Table 1.

**Table A3:** Dynamic patterns of symbolic encoding with two thresholds (Method 2: the 2.5% and 97.5% sample quantiles) for sequences of length k=3 and k=4 within the pre-war in Ukraine and the war periods (for the 15 analyzed stock market indices).

Country	The Pre-War in Ukraine Period				The War in Ukraine Period			
	All Sequences for *k* = 3	The Sequence (1,1,1)	All Sequences for *k* = 4	The Sequence (1,1,1,1)	All Sequences for *k* = 3	The Sequence (1,1,1)	All Sequences for *k* = 4	The Sequence (1,1,1,1)
France	256	218 (85.2%)	255	222 (87.1%)	257	207 (80.5%)	256	213 (83.2%)
U.K.	251	214 (85.3%)	250	214 (85.6%)	249	205 (82.3%)	248	203 (81.9%)
Germany	253	214 (84.6%)	252	222 (88.1%)	257	203 (79.0%)	256	213 (83.2%)
Finland	249	210 (84.3%)	248	218 (87.9%)	249	199 (79.9%)	248	212 (85.5%)
Norway	250	215 (86.0%)	249	212 (85.1%)	253	206 (81.4%)	252	199 (79.0%)
Turkey	247	213 (86.2%)	246	216 (87,8%)	248	206 (83.1%)	247	207 (83.8%)
Poland	249	213 (85.5%)	248	213 (85.9%)	251	202 (80.5%)	250	203 (81.2%)
Hungary	250	212 (84.8%)	249	224 (89.9%)	252	202 (80.2%)	251	218 (86.9%)
Czechia	249	208 (83.5%)	248	222 (89.5%)	252	195 (77.4%)	251	216 (86.1%)
Romania	249	210 (84.3%)	248	219 (88.3%)	250	198 (79.2%)	249	213 (85.5%)
Bulgaria	243	204 (83.9%)	242	218 (90.1%)	246	192 (78.0%)	245	213 (86.9%)
Lithuania	247	210 (85.0%)	246	221 (89.8%)	249	199 (79.9%)	248	214 (86.3%)
Estonia	250	218 (87.2%)	249	222 (89.2%)	252	210 (83.3%)	251	215 (85.7%)
Latvia	246	207 (84.1%)	245	222 (90.6%)	252	196 (77.8%)	251	215 (85.7%)
Slovakia	247	210 (85.0%)	246	188 (76.4%)	220	198 (90.0%)	219	178 (81.3%)

Notation as in Table 1.

## Appendix B. Symbol–Sequence Histograms: Additional Figures

Figure A1, Figure A2, Figure A3 and Figure A4 present comparative analyses of symbol–sequence histograms based on Definition 2 and Method 1 with two thresholds (5% and 95%) within two pairs of turbulence periods: (1) the pre-COVID-19 and the COVID-19 pandemic periods and (2) the pre-war and the war periods in Ukraine. Examples of appropriate histograms for six stock markets are reported; however, the histograms for the remaining markets are very similar and they are available upon request (due to space restrictions). The empirical results visualized by histograms are documented in Table 5 and Table 6 and they are unambiguous for all investigated indices. The main evidence shows that the sequence (1,1,1) (see Table 4) is the most frequently observed. This sequence means that three successive daily stock index returns are not extremely high or low, but they lie between the sample quantile thresholds of $\theta_{1}=5\%$ and $\theta_{2}=95\%$ (Definition 2). Moreover, the histograms document that the number of zero-frequency sequences is relatively high for all indices and all investigated periods.

## Appendix C. The Modified Shannon Entropy: Additional Comparative Results

Table A4 and Table A5 report additional comparative findings of Method 1 (the 5% and 95% sample quantiles) for sequences of length k=4 and k=5 within two pairs of turbulence periods: (1) the pre-COVID-19 and the COVID-19 pandemic periods (Table A4) and (2) the pre-war and war periods in Ukraine (Table A5). The findings are homogenous and they document that the choice of the sequence length *k* is a minor problem as the results for k=4 and k=5 are similar to those for k=3 (see Table 8 and Table 10). This evidence once again confirms that Method 1 with two 5% and 95% quantile thresholds (for sequences of length k=3,4,5) is worth a recommendation in financial time series applications.

**Table A4:** The modified Shannon entropy based on symbolic encoding with two thresholds given by Definition 2. Comparative empirical findings of Method 1 (the 5% and 95% sample quantiles) for sequences of length k=4 and k=5 within the pre-COVID-19 and COVID-19 periods for the 15 analyzed stock market indices.

Country	The Modified Shannon Entropy Based on Symbolic Encoding with Two Thresholds					
	Definition 2, Method 1, *k* = 4			Definition 2, Method 1, *k* = 5		
	Pre-COVID-19	COVID-19	Change	Pre-COVID-19	COVID-19	Change
France	0.438	0.403	−0.035 ↓	0.476	0.433	−0.043 ↓
U.K.	0.450	0.397	−0.053 ↓	0.493	0.422	−0.071 ↓
Germany	0.450	0.428	−0.022 ↓	0.495	0.453	−0.042 ↓
Finland	0.453	0.397	−0.056 ↓	0.491	0.419	−0.072 ↓
Norway	0.448	0.400	−0.048 ↓	0.485	0.435	−0.050 ↓
Turkey	0.468	0.412	−0.056 ↓	0.503	0.444	−0.059 ↓
Poland	0.476	0.415	−0.061 ↓	0.517	0.449	−0.068 ↓
Hungary	0.434	0.395	−0.039 ↓	0.478	0.425	−0.053 ↓
Czechia	0.444	0.391	−0.053 ↓	0.489	0.419	−0.070 ↓
Romania	0.449	0.374	−0.075 ↓	0.489	0.399	−0.090 ↓
Bulgaria	0.406	0.383	−0.023 ↓	0.461	0.422	−0.039 ↓
Lithuania	0.395	0.362	−0.033 ↓	0.437	0.393	−0.044 ↓
Estonia	0.429	0.374	−0.055 ↓	0.480	0.404	−0.076 ↓
Latvia	0.434	0.401	−0.033 ↓	0.462	0.448	−0.014 ↓
Slovakia	0.470	0.408	−0.062 ↓	0.514	0.443	−0.071 ↓

Notation as in Table 1.

**Table A5:** The modified Shannon entropy based on symbolic encoding with two thresholds given by Definition 2. Comparative empirical findings of Method 1 (the 5% and 95% sample quantiles) for sequences of length k=4 and k=5 within the pre-war in Ukraine and the war periods for the 15 analyzed stock market indices.

Country	The Modified Shannon Entropy Based on Symbolic Encoding with Two Thresholds					
	Definition 2, Method 1, *k* = 4			Definition 2, Method 1, *k* = 5		
	Pre-War	War	Change	Pre-War	War	Change
France	0.460	0.456	−0.004 ↓	0.502	0.506	0.004 ↑
U.K.	0.482	0.515	0.033 ↑	0.532	0.546	0.014 ↑
Germany	0.505	0.464	−0.041 ↓	0.539	0.500	−0.039 ↓
Finland	0.476	0.460	−0.016 ↓	0.512	0.506	−0.006 ↓
Norway	0.505	0.473	−0.032 ↓	0.555	0.509	−0.046 ↓
Turkey	0.449	0.432	−0.017 ↓	0.485	0.470	−0.015 ↓
Poland	0.495	0.477	−0.018 ↓	0.547	0.524	−0.023 ↓
Hungary	0.464	0.399	−0.065 ↓	0.505	0.431	−0.074 ↓
Czechia	0.554	0.402	−0.152 ↓	0.589	0.444	−0.145 ↓
Romania	0.464	0.447	−0.017 ↓	0.506	0.480	−0.026 ↓
Bulgaria	0.522	0.423	−0.099 ↓	0.566	0.474	−0.092 ↓
Lithuania	0.474	0.410	−0.064 ↓	0.511	0.451	−0.060 ↓
Estonia	0.424	0.446	0.022 ↑	0.464	0.495	0.031 ↑
Latvia	0.501	0.387	−0.114 ↓	0.549	0.417	−0.132 ↓
Slovakia	0.481	0.467	−0.014 ↓	0.527	0.528	0.001 ↑

Notation as in Table 1.
